# Arabinoxylan‐enriched fraction from durum wheat meal modulates structure, texture, nutritional and sensory properties of extruded snacks

**DOI:** 10.1002/jsfa.71033

**Published:** 2026-09-07

**Authors:** Vittoria Latrofa, Aleksei Kaleda, Carlo Porfido, Giusy Rita Caponio, Giuseppe Natrella, Davide De Angelis, Roberto Terzano, Antonella Pasqualone

**Affiliations:** ^1^ Department of Soil, Plant and Food Science (DISSPA) University of Bari Aldo Moro Bari Italy; ^2^ TFTAK AS Tallinn Estonia; ^3^ Department of Bioscience, Biotechnology and Environment University of Bari Aldo Moro Bari Italy

**Keywords:** arabinoxylans, extruded snacks, durum wheat by‐products, low‐moisture extrusion, X‐ray microtomography

## Abstract

**BACKGROUND:**

Arabinoxylans (AX), abundant in wheat bran, represent a valuable yet underutilized fraction of cereal milling by‐products. In this study, an AX‐enriched fraction obtained from durum wheat meal (DWM), produced from defatted durum wheat germ and debranning fractions, was incorporated at increasing levels (0–25%) into pea starch concentrate (PSC) and processed by low‐moisture extrusion to produce expanded snacks.

**RESULTS:**

Progressive replacement of PSC with DWM significantly affected product structure. Expansion ratio (%) decreased from 6.88 ± 0.63 to 1.67 ± 0.36, accompanied by a reduction in specific volume, mainly due to the higher fiber content and lower amylose of DWM. X‐ray microtomography revealed significant modifications in internal architecture, with reduced expansion and thinner solid matrices. These structural changes affected texture as hardness increased from 2325 ± 164 g (control) to 9577 ± 920 g (25% DWM), while crispness decreased from 41.33 ± 4.23 g to 8.40 ± 1.95 g. DWM incorporation improved predicted glycemic index and reduced water holding capacity, while oil holding capacity remained unchanged. Sensory analysis showed increased roastiness, bitterness, and astringency, typical of wholegrain products, without off‐odors or off‐flavors.

**CONCLUSION:**

These results show that durum wheat milling by‐products can be successfully upcycled into AX‐enriched extruded snacks, improving nutritional value while maintaining acceptable structural quality. © 2026 The Author(s). *Journal of the Science of Food and Agriculture* published by John Wiley & Sons Ltd on behalf of Society of Chemical Industry.

## INTRODUCTION

Arabinoxylans (AX) are complex polysaccharides, the backbones of which are composed of d‐xylopyranosyl units linked by β‐1,4 bonds, substituted with α‐l‐arabinofuranosyl residues at position 2 and/or 3.^1^, ^2^ They are major structural components of cereal cell walls and are found in grains such as wheat, rye, barley, oat, rice, and corn.^1^, ^3^ In wheat, AX account for approximately 4–8% of the kernel and are mainly concentrated in the bran layers generated during debranning and milling operations.^2^ Most wheat AX (70–80%) are water‐unextractable due to their association with other cell wall components.^1^, ^2^ Interest in AX has increased because of their physiological effects, including modulation of postprandial glycaemia and cholesterol response, improved mineral absorption, prebiotic activity, and potential hepatoprotective and immunomodulatory properties.^3^

Conventional recovery of AX from wheat bran generally relies on alkaline or enzymatic extraction processes to solubilize water‐unextractable fractions. However, these approaches require large amounts of water, chemicals, enzymes, and energy.^2^ As an alternative, dry fractionation technologies such as air classification can produce AX‐enriched fractions through fine milling followed by particle separation based on size and density, avoiding the use of solvents and reducing process impacts.^2^, ^4^

AX‐rich ingredients have been investigated in several food applications, including bread, confectionery products, pastries, and beverages.^3^ Their incorporation into expanded snacks is of particular interest because these products are typically characterized by high starch digestibility, high energy density, and limited nutritional value.^5^ The use of fiber‐rich cereal fractions or legume‐derived ingredients has therefore been proposed to improve the nutritional profile of expanded snacks while maintaining acceptable technological properties.^5^, ^6^

Expanded snacks are commonly produced by extrusion cooking, a process in which hydrated raw materials undergo intense thermomechanical treatment before expansion at the die exit due to the rapid release of pressure and vaporization of water.^5^, ^7^ The resulting products are characterized by low density, porous structure, crispy texture, and roasted flavor notes.^8^ Previous studies have shown that the incorporation of bran fractions or AX‐rich ingredients can substantially modify the structural and sensory properties of extruded products. Fleischman *et al*.^9^ reported that wheat bran addition increased antioxidant activity but reduced expansion in extruded snacks. Reis and Abu‐Ghannam^10^ observed that brewer's spent grain increased AX content and lowered the predicted glycemic index of extruded formulations. Grasso^7^ further highlighted that, despite the growing interest in incorporating cereal by‐products into extrusion processes, their effects on product texture and sensory quality remain insufficiently understood. Two main gaps can be identified in the available literature. First, most studies have evaluated the incorporation of whole bran^11^ or whole ingredients containing AX^10^ into expanded snacks whereas, in other cases, purified AX^12^ have been used. From this perspective, the use of dry‐fractionated AX‐rich ingredients, particularly those obtained from durum wheat, remains largely unexplored despite the industrial relevance of durum wheat milling by‐products. Second, quantitative microstructural characterization of expanded snacks has received limited attention, although it is essential for explaining the development of texture and its relationship with sensory properties.

Investigating the effects of durum wheat AX‐enriched fractions on the microstructure, texture, nutritional value, and sensory properties of extruded snacks may improve the understanding of their technological functionality while supporting the valorization of durum wheat by‐products. Therefore, the objective of this study was to develop an AX‐enriched expanded snack using dry‐fractionated durum wheat (*Triticum turgidum* subsp. *durum* (Desf.)) meal as an ingredient. Specifically, this by‐product was selected for its high content in AX and was produced through a dry fractionation process (air classification) of durum wheat meal (DWM; i.e., defatted durum wheat germ and debranning fractions), which is further separated by air classification into a protein‐rich fraction and a fraction rich in polysaccharides and arabinoxylans. In this study, the AX‐enriched fraction was used for snack production in combination with pea starch. The effects of its incorporation on the microstructural, textural, nutritional, and sensory properties of the extruded snacks were then evaluated.

## MATERIALS AND METHODS

### Materials

The AX fraction obtained thought the dry fractionation of defatted DWM was provided by Casillo Next Gen Food S.r.l. (Corato, Italy). The production process included fine milling followed by air classification. The dry‐fractionated pea starch concentrate (PSC) was obtained from Aloja‐Starkelsen (Aloja, Latvia). The two ingredients were analyzed to determine the content of protein, starch, ash, and lipid, as described in the section ‘Chemical determination of ingredients’ and arabinoxylans as described in the section ‘Amylose and arabinoxylan determination in powder blends’, below. Fiber content was obtained from the producer. The nutritional composition of DWM was 11.27 proteins, 0.52 fats, 20.27 starch, 3.29 ash, 13.34 fiber, 12.34 remaining carbohydrates, and 38.97 AX g 100 g^−1^ (dry matter basis), whereas PSC had 11.50 proteins, 1.91 fats, 8.86 fiber, 70.36 starch, and 7.37 remaining carbohydrates g 100 g^−1^ (dry matter basis).

### Chemical determination of the ingredients

Protein content (N × 6.25) of the raw materials was determined according to the reported method.^13^ The lipid content was carried out using a Randall apparatus (SER 148 extraction system, Velp Scientifica S.r.l., Usmate Velate, Italy) according to the method AOAC 945.38F,^13^ with diethyl ether as extraction solvent. Ash was determined as described in the method AOAC 923.03.^13^ Total starch content was determined using a total starch assay kit (Megazyme Ltd, Bray, Ireland), following method AOAC 996.11.^13^, ^14^

### Extrusion cooking

Extrusion cooking was performed using a co‐rotating intermeshing twin‐screw extruder KETSE 20/40 (Brabender GmbH, Duisburg, Germany), using conditions determined in preliminary trials. The temperature profile was set to 40/65/105/135/146/154 °C, the screw speed to 300 rpm, the total mass flow rate to 5.2 kg h^−1^, and the total moisture to 16.0%. These conditions were optimized for the reference PSC sample to obtain a good texture of the product: well expanded, soft, crunchy, and with a uniform shape, judged by informally evaluating the product during extrusion. Lower moisture, higher screw speed and temperature resulted in product burning, while the opposite settings resulted in insufficient puffing and hard texture.

The specific mechanical energy (SME) was calculated as described in Latrofa *et al*.^15^ Before extrusion, each powder was calibrated to determine the powder mass flow rate in the volumetric feeder. Water was added using a calibrated peristaltic pump. The diameter of the die hole was 1.6 mm. The extrudates were collected after the process stabilized – that is, when the temperature, die pressure, and screw torque, recorded every second by the extruder, were constant. The samples were dried and cooled in the air, and then packed into low‐density polyethylene (LDPE) zip‐lock bags and stored at room temperature.

### Amylose and arabinoxylan determination in powder blends

Amylose was determined in the raw materials and in the snacks using the assay kit Amylose/Amylopectin (Megazyme), which is based on the precipitation of amylopectin using concanavalin‐A, according to the information given by the manufacturer. The concentration of amylose was calculated as the ratio of glucose oxidase peroxidase (GOPOD) absorbance of the supernatant of the concanavalin A‐precipitated sample to the absorbance of the total starch sample (Megazyme), measured at 510 nm using a Cary 60 UV–visible spectrophotometer (Agilent Technologies, Santa Clara, CA, USA).

The AX content was determined in the raw materials and in the snacks using a method that measures pentosanes content within samples.^2^ Briefly, 5 mg of a powder was suspended in 2 mL of water in a glass tube. Subsequently, after mixing on a vortex mixer, 10 mL of extraction solution (110 mL of glacial acetic acid, 2 mL of hydrochloric acid, 5 mL of 20% (w/v) phloroglucinol in absolute ethanol, and 1 mL of 1.75% (w/v) glucose in water) were added. After stirring, the glass tubes were placed in a 100 °C water bath for 25 min and stirred twice during the incubation time. Subsequently, the glass tubes were cooled down in flowing cold water to stop the reaction, and the absorbance was read at 510 and 552 nm. The absorbance read at 510 nm was subtracted from the absorbance read at 552 nm to eliminate the influence of hexose sugars. The AX content was calculated using a calibration curve (*R* ^2^ = 0.998), with a series of standard solutions of d‐(+)‐xylose at 0, 0.25, 0.1, 0.05, and 0.075 mg mL^−1^.

### Physical and physicochemical properties

The expansion ratio (ER) was calculated by dividing the diameter of the extruded snack, measured using a caliper, by the diameter of the extruder die hole.^8^ Water holding capacity (WHC), water solubility index (WSI), and oil holding capacity (OHC) were determined as described by Kaleda *et al*.^16^ Specific volume was measured as described in Latrofa *et al*.^15^ according to the rapeseed displacement AACC method 10‐05.01.^17^

Texture profile analysis (TPA) was performed following the method used by Paula and Conti‐Silva,^18^ with some modifications. A TA.XTplusC Texture Analyzer (Stable Micro‐Systems, Godalming, UK) equipped with a compression plate of 75 mm in diameter was used. Briefly, three extrudates were cut to a length of 3 cm and compressed once under the following conditions: 3 mm s^−1^ pre‐test, 10 mm s^−1^ test speed, 60% compression, 50 kg load cell. During compression, the snack breaks multiple times, and this creates sharp drops on the hardness line. The analysis counted the number of peaks formed due to this process. The maximum hardness (g) and the number of peaks were calculated using the texture analyzer software. The peak count was related to the crispness of the snack.

The color of the extrudates in the CIELAB color space was measured using an NS810 spectrophotometer (3nh Shenzhen Threenh Technology Co., Ltd, Shenzhen, China) set to illuminant D65 and 2°. Lightness (L*, from black to white), redness (a*, from green to red), and yellowness (b*, from blue to yellow) were evaluated.

### Micro‐computed tomography 3D analysis

A SkyScan 1272 micro‐computed tomography (micro‐CT) system (Bruker Nano GmbH, Berlin, Germany) was used to investigate the internal structure and various morphometrical parameters of PSC and DWM 5–25% samples.^19^ As a non‐destructive technique, micro‐CT emerges as an ideal approach to obtain qualitative and quantitative information for quite brittle specimens like these, compared to other microscopies that require sample mechanical sectioning or other kinds of destructive preparations. Moreover, the almost cylindrical shape of the extrudates represents an optimal condition for micro‐CT analysis. The SkyScan instrument was equipped with a tungsten microfocus source (60 kV, 160 μA, <5 μm spot size) and a 16 Mp charge‐coupled device detector. For all samples, the acquisition settings were pixel size 2.5 μm, rotation step 0.2° (from 0° to 180°), and exposure time of 900 ms. To improve data quality, flat field correction, averaging three frames and random movement of 10 pixels, were used during acquisition. Angular projections were reconstructed using NRecon software version 1.6.10.4, while CT Analyzer software (version 1.15.4.0 + 4, Bruker micro‐CT) and CTvox (version 3.1.1 r1191, Bruker micro‐CT) were used for measuring quantitative parameters and for 3D rendering, respectively.

### *In vitro* starch hydrolysis and predicted glycemic index

*In vitro* starch hydrolysis was determined in accordance with Caponio *et al*.^20^ with some modifications, simulating *in vivo* starch digestion. Hydrolyzed starch was measured by quantifying the amount of released glucose using the d‐glucose assay kit (GOPOD format; Megazyme). Starch was calculated as glucose (mg) × 0.9 (conversion factor). Simulated digests were dialyzed for 16 h using a membrane with a cut‐off of 12 400 Da. Aliquots of dialysate, containing free glucose and partially hydrolyzed starch, were sampled after 3 h and at the end of digestion, after 16 h. These were further treated with amyloglucosidase. Then, free glucose was quantified using the above‐mentioned enzyme‐based kit and finally converted into hydrolyzed (digested) starch in the snack samples. White wheat bread was used as a control to estimate the hydrolysis index (HI = 100). The predicted glycemic index (pGI) was calculated using the equation pGI = 0.549 × HI + 39.71, as described by Caponio *et al*.^21^ Each sample was analyzed in triplicate.

### Antinutritional compounds

Total phytate content of the raw materials and snacks was determined using the phytic acid assay kit K‐PHYT 05/07 (Megazyme), according to the manufacturer's instructions. Oligosaccharides (verbascose, stachyose, raffinose, and sucrose) in the snacks were determined according to Squeo *et al*.,^22^ with some modifications. Briefly, the determination was made using high‐performance liquid chromatography (HPLC; Agilent Technologies), equipped with a refractive index detector (RID 1260). Milled snacks (5 mg) were dispersed in 10 mL of deionized water, stirred for 5 min, and filtered through 0.22 μm cellulose acetate filters. The HPLC separation was performed isocratically at a flow rate of 0.8 mL min^−1^ through a 300 × 7.8 mm cation‐exchange column (Rezex RCM column, Ca^2+^, 8 μm, Phenomenex, Torrance, CA, USA) maintained at 80 °C, with deionized water used as a mobile phase. In order to identify the oligosaccharides, the retention time of the peak was compared with the corresponding standard (Merck KGaA, Darmstadt, Germany); in order to quantify them, a calibration curve for each oligosaccharide was obtained.

### Sensory analysis

Descriptive sensory analysis was conducted at TFTAK AS (Tallinn, Estonia) by an internal sensory panel. The assessments were made individually in isolated testing booths in a dedicated sensory room (in accordance with ISO 2007^23^). Ten participating assessors (average age 33 ± 9) had previous training and experience in evaluating extrudate snacks and gave written consent to take part in the sensory analysis. Participation in the study was voluntary, and panelists could withdraw from the test at any time. The selected panelists were in good health, had no known allergies to the components, and were informed before the analysis about the purpose and procedures of the study. Participants were assured of the confidentiality of their data.

This study was conducted in accordance with the ethical principles outlined in The Code of Ethics of the World Medical Association (Declaration of Helsinki) and the Principles of Academic Ethics by Tallinn University of Technology. The research followed the protocols designed to protect the rights, privacy, and welfare of participants.

Sensory analysis was conducted in two replicate sessions, with each sample presented once per session. A 2 h break was included between the sessions to mitigate sensory fatigue. All samples were coded with random three‐digit codes, and the order of assessment was randomized for each assessor according to Williams' Latin Square design to reduce the presentation effect.^24^ Samples were served to assessors at room temperature (21 °C) in clear 40 mL plastic cups and presented in sequentially monadic order. Quantitative descriptive analysis used a 10‐point structured scale, where 0 is 'none', 1 is 'very weak', 5 is 'moderate', and 9 is 'very strong'.

The sensory analysis was conducted according to an internal sensory protocol, which focused mainly on three modalities: odor, taste, and texture. For odor and taste, overall intensity and raw material intensity (such as cereals/legumes), sourness, sweetness, and roastiness were the attributes to be evaluated. Roastiness was used as a generic term for overall toasted nuances, and assessors could also evaluate off‐odor and off‐taste as separate attributes when detected. All texture attributes were assessed orally. Hardness indicated the force required to compress the sample between the molar teeth; crispness was defined as the extent to which the product crumbled/broke, together with noise generated when biting through; puffiness was identified as the feeling of a light and porous composition when biting through; and adhesiveness was described as the extent to which the sample adheres to the teeth when chewing the sample. Data collection was carried out using sensory software RedJade version 6.1 (RedJade Sensory Solutions LLC, Martinez, CA, USA).

### Statistical analysis

All data are expressed as means with standard deviations. ER, WHC, WSI, OHC, antinutritional factors, arabinoxylans, amylose, color, HI, and pGI were measured in triplicate, TPA in eight replicates, and sensory attributes in duplicate by ten assessors. The means of the samples were analyzed using a one‐way ANOVA followed by Tukey's HSD (Honestly Significant Difference) test for multiple comparisons at a significance level *α* = 0.05 using Minitab 19 Statistical Software (Minitab Inc., State College, PA, USA). Pearson's correlation was carried out using the same software. The figures were created using GraphPad Prism version 9 (GraphPad Software, San Diego, CA, USA).

## RESULTS AND DISCUSSION

### Visual appearance of the extruded snacks and extruder operating performance

The recorded parameters during low‐moisture extrusion of the snacks are presented in Fig. 1, while Fig. 2 shows the visual appearance of the snacks produced at different concentrations. Extruder operating performance is driven by physicochemical reactions of macromolecules under specific conditions, which influence structure and puffing formation. Differences in expansion between fiber and starch are due to variations in water absorption, melt viscoelasticity at the die exit, and bubble formation during expansion, resulting in different extrusion responses.^15^, ^25^

**Figure 1:**
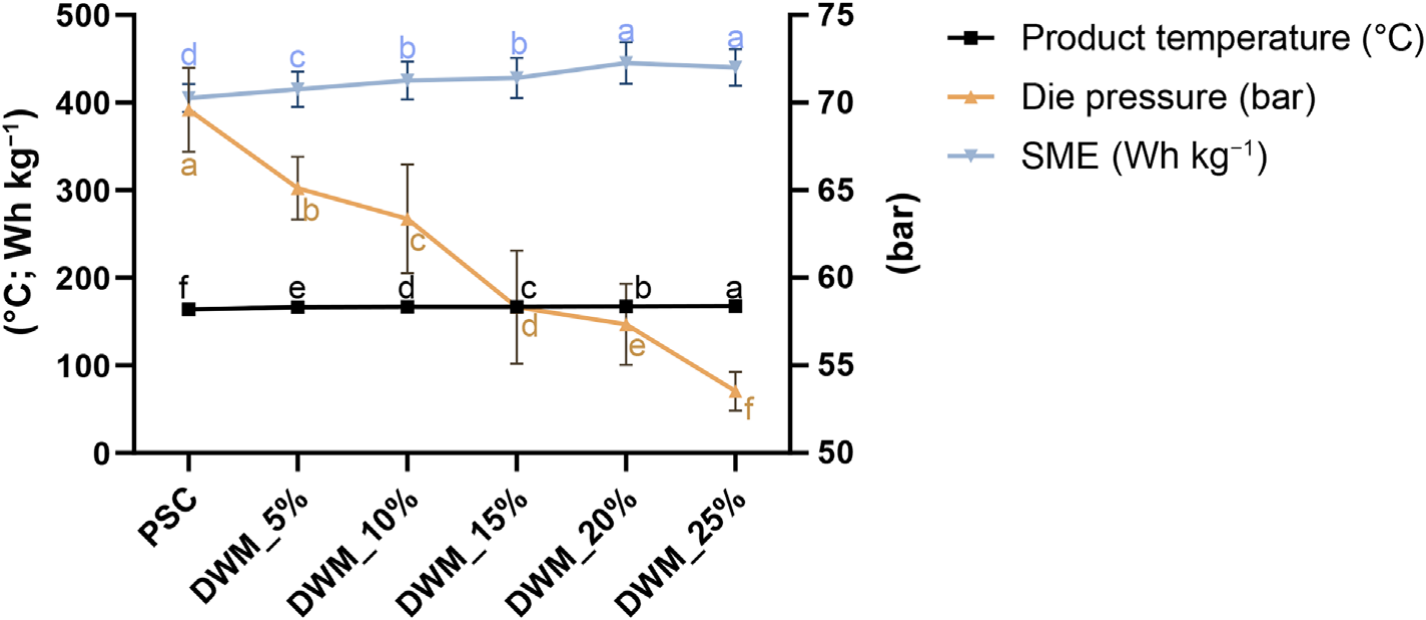
Extruder operating performance relative to each snack formulation. PSC, pea starch concentrate; DWM, durum wheat meal arabinoxylan fraction; SME, specific mechanical energy. Data are expressed as mean ± SD (*n =* 181). Different letters for the same parameter indicate significant differences according to Tukey's HSD test (*P* ≤ 0.05).

**Figure 2:**
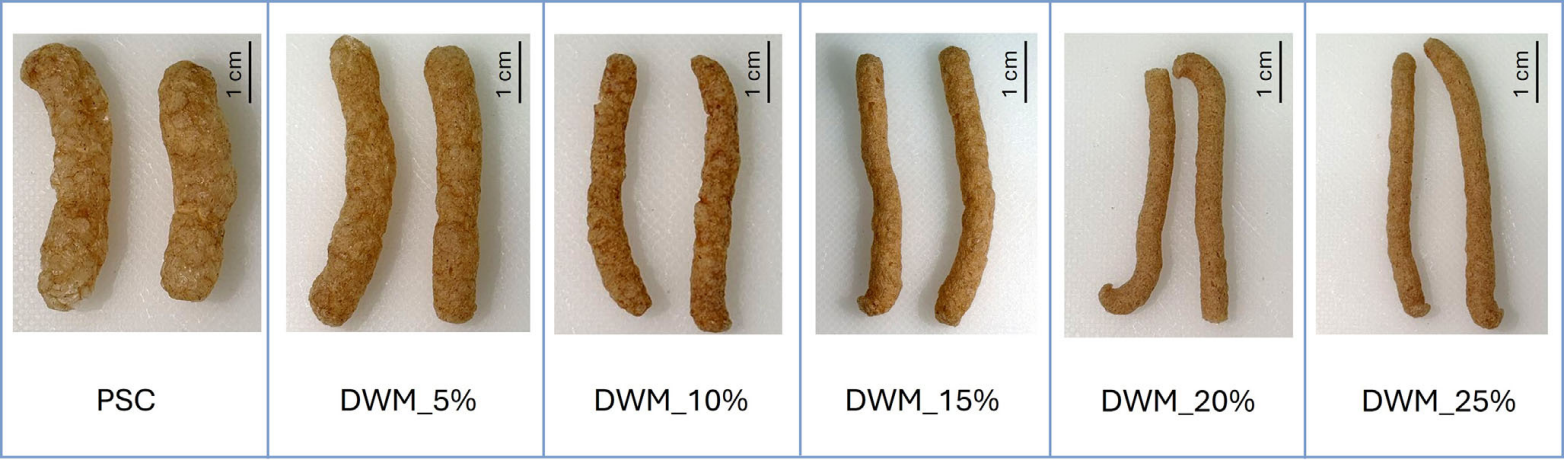
Visual appearance of the extruded snacks produced from a blend of pea starch concentrate (PSC) and durum wheat meal arabinoxylan fraction (DWM) blends.

Increasing DWM ratio in the formulation resulted in higher melt temperature at the die and SME, accompanied by a decrease in the die pressure. Martin *et al*.^26^ reported a correlation between SME and amylopectin content, suggesting that amylopectin, being a large molecule, has a greater surface area compared to amylose, causing an increase in viscosity in the first part of the barrel and thus higher internal friction and SME, corroborating the results described in the next section. Higher friction in the middle of the extruder barrel led to an increase in melt temperature at the die, ultimately resulting in a decrease in viscosity at the end of the barrel at the die. The decrease in die pressure due to lower viscosity^27^ at the exit could have been related to the lower expansion of the samples containing DWM (see the section ‘Physical and physicochemical properties’, above).

### Microstructure and morphometry of expanded snacks

The results of the X‐ray micro‐CT analysis are presented in Fig. 3 and Table 1. DWM caused relevant changes in both extrudates' morphology and morphometry, particularly remarkable for concentrations of 10% and above, in comparison to the PSC sample. First, increasing DWM affected the snacks' shape, making them progressively more cylindrical, as visible from the side views in Fig. 3. The surface morphology of extrudates changed from the smooth and bubbling appearance of PSC to the rough and barky appearance of DWM recipes, likely caused by the increasing presence of fibers on the surface. Transversal and longitudinal views in Fig. 3 show a huge transformation of pore structure after DWM substitutions. Indeed, when the DWM percentage increases, pores become smaller and more homogeneous in both shape and size. PSC looks quite irregular inside, with bubbles and inner structures showing evident deformations arising from expansion and subsequent collapse caused by cooling and/or decompression processes. On the other hand, the internal structure of DWM snacks appears more regular, with almost spherical pores and without any evidence of structural collapse. Morphometry results (Table 1) quantitatively confirmed these differences among the formulations investigated. The object volume (OV) indicates the volume occupied by the solid fraction of the expanded snack within the total volume of interest (TV) examined, which also includes the pore spaces. As expected, an increase in DWM caused a reduction in both TV (already evident from a simple visual inspection) and OV values for a linear unit of extrudate, similarly to what was observed for the specific volume and expansion ratio parameters (see the section ‘Physical and physicochemical properties’, above). This result is consistent with the literature, which reports that the presence of fiber in a starch‐based formulation reduces the expansion of the product.^28^, ^29^, ^30^ Moreover, these results confirm the findings of the texture analysis and of the expansion ratio. Karkle *et al*.^29^ explained that fiber is incompatible with starch, and during the shearing in the extruder die, it hinders extensibility, expansion, and the rupture of air cells. Our results show that an increment of 5% of DWM was sufficient to reduce the OV of the snack by approximately 40%, while DWM_10% reduced the OV by about 60%. Conversely, at higher DWM concentrations, OV remained similar to that at 10%. Along with decreasing OV, total porosity also decreased, from 94% of PSC to 85% of DWM_25%. Object surface‐to‐volume ratio (OS/OV) was also affected by the addition of DWM in the snack, with increasing values for increasing DWM in the formulation. This result is connected to the concurrent decrease in OV and the increase in the object total surface, which is due to the much higher number of pores and vesicles formed at higher levels of DWM (Fig. 3). Similar findings were described by Dey *et al*.^31^ while incorporating fiber into extruded products. Dey *et al*.^31^ explained that the low expansion of the extrudates could be related to fiber aggregation in the matrix. In this regard, the decrease in the structure separation parameter (St.Sp.) suggested a more compact structure, while higher values indicated larger and connected air bubbles, as visible in Fig. 3 for the control sample PSC and still evident for DWM_5%.^19^ In fact, St.Sp. estimates the average distance separating object structures – in other words, the distance between air bubble walls in the snack.^19^ Finally, the decrease in structure thickness appears coherent with the results described so far, arising from both an overall reduction in the snack dimension and an enhanced number of pores, which hinder the formation of more but less‐thick solid structures (i.e., cell walls). Nevertheless, an improvement in the extrusion conditions should be explored in order to try to improve the expansion of the samples with higher DWM concentration.

**Figure 3:**
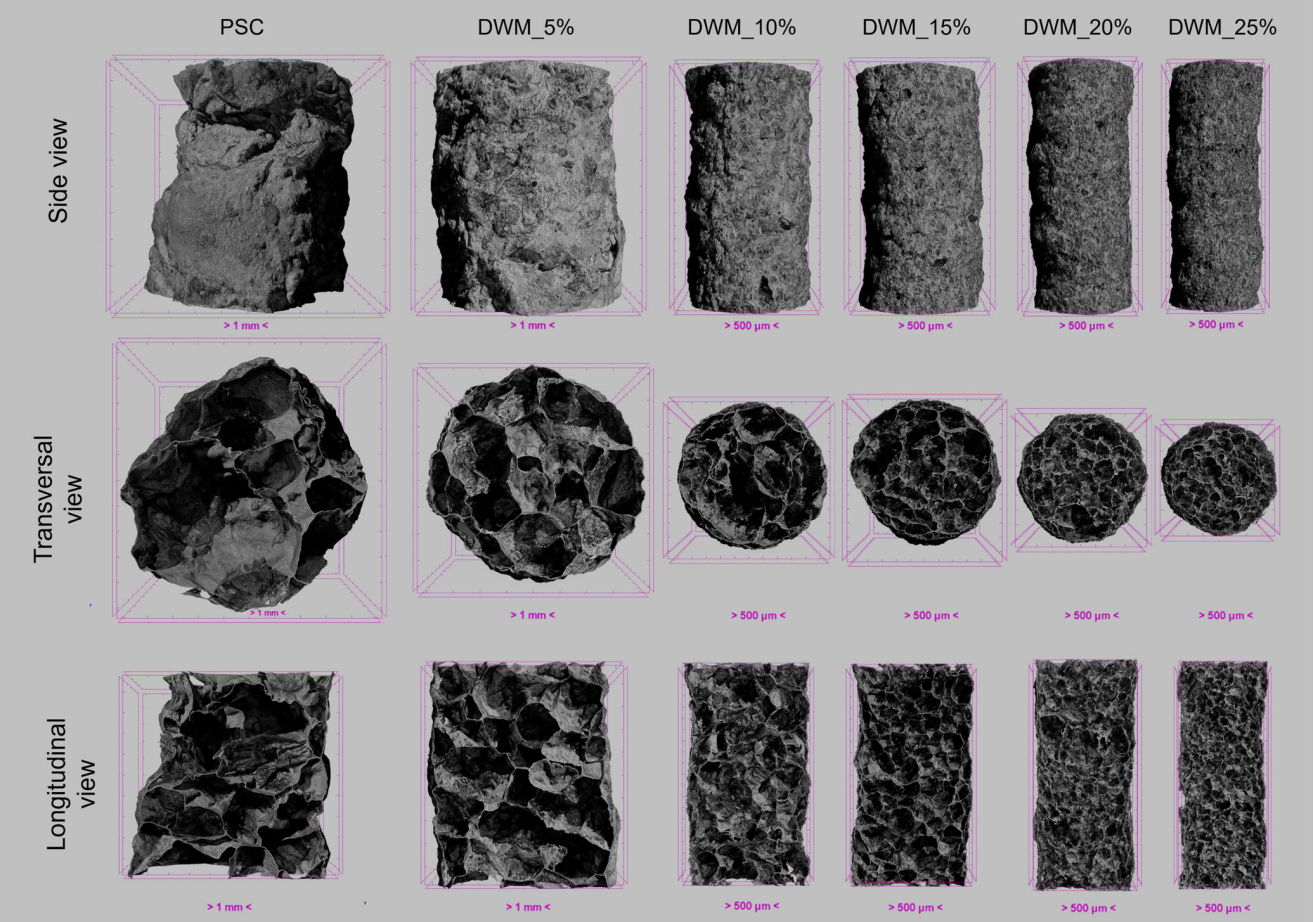
Micro‐CT 3D renderings of pea starch concentrate (PSC) and durum wheat meal arabinoxylan fraction (DWM) extrudate samples. The micrographs portray samples of 10 mm height. In the top figure, external side views show superficial features of the snacks; in the bottom part of the figure, virtual sections show internal morphology and pore structure in transversal and longitudinal views, respectively.

**Table 1:** X‐ray microtomography analysis of morphometry of snacks

Sample	TV (mm^3^)	OV (mm^3^)	OV/TV (%)	St.Th. (μm)	St.Sp. (μm)	OS/OV (mm^−1^)	Po.V. (mm^3^)	TP (%)
PSC	768.1	43.1	5.6	55.5	1215.5	3.2	725.0	94.4
DWM_5%	569.3	24.8	4.4	32.7	1031.6	4.4	544.5	95.6
DWM_10%	245.8	18.1	7.4	31.2	531.5	8.7	227.7	92.6
DWM_15%	231.7	21.9	9.4	32.4	436.7	10.6	209.8	90.6
DWM_20%	157.2	16.9	10.8	26.0	280.8	16.3	140.3	89.2
DWM_25%	121.2	18.7	15.4	28.0	412.7	19.7	102.5	84.6

Reported data refer to 10 mm length samples. Snacks were produced from pea starch concentrate (PSC) and durum wheat meal arabinoxylan fraction (DWM). OSOV, object surface‐to‐object volume ratio; OV, object volume; OV/TV, percent object volume; Po.V., total volume of pore space; St.Sp., structure separation (mean); St.Th., structure thickness (mean); TP, total porosity percent; TV, total volume.

### Amylose and arabinoxylan content

Figure 4 shows the amylose and AX content of the snacks. The amylose content decreased as the DWM content increased in the formulation. These results were expected, considering that the amylose content was 40.88% and 23.21% in initial PSC and DWM powders, respectively. Previously, other authors^32^, ^33^ reported an amylose content of 41.1% and 27.2% in PSC^32^ and durum wheat,^33^ respectively. During extrusion cooking, the high temperature, water, and mechanical treatment lead to changes in the semi‐crystalline regions of starch which, due to gelatinization, are destroyed and converted into amorphous ones.^34^ Amylose content can affect different properties of extruded snacks, such as digestibility, texture, and expansion. From a technological point of view, high amylose content facilitates the gelatinization process during extrusion, resulting in more expanded and less hard products.^35^ This hypothesis was confirmed by the ER and the texture profile analysis (see section ‘Instrumental texture and color’, below) and further supported by X‐ray micro‐CT analysis, which showed that snacks containing higher DWM levels exhibited a more compact cellular structure, with lower porosity and smaller pores. The incorporation of DWM into snacks resulted in an increase in AX. The use of by‐products to produce extruded snacks enriched in AX was previously studied by Reis and Abu‐Ghannam.^10^ Those authors used brewer's spent grain in cereal‐based snacks and showed an increase in AX compared to the control produced from blends of rice flour and wheat semolina. Of note, wheat AX have been linked to functional properties with potential beneficial implications for human health. Evidence suggests their role in modulating glycemic response and lipid metabolism, as explained in the section 'Predicted glycemic index and antinutritional compounds', below, as well as prebiotic activities.^36^

**Figure 4:**
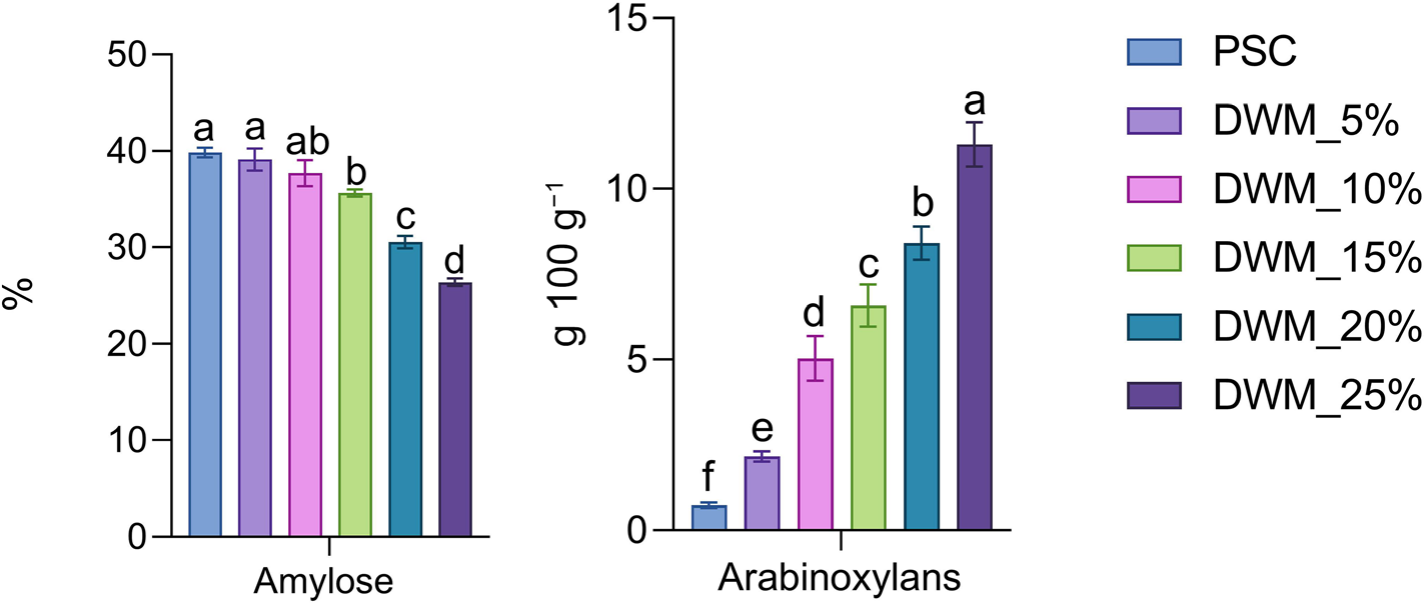
Amylose and arabinoxylan content in the extruded snacks produced from a blend of pea starch concentrate (PSC) and durum wheat meal arabinoxylan fraction (DWM). The values represent means of triplicates ± SD. Different letters in the same column indicate significant differences according to one‐way ANOVA, followed by Tukey's HSD test (*P* ≤ 0.05).

### Physical and physicochemical properties

The physical and physicochemical properties of snacks are reported in Table 2. ER was reduced by increasing the DWM content in the snack formulation. ER describes the puffiness of extrudates, which is related to a softer and less dense structure resulting from the presence of few, but large, air cells.^9^, ^37^ Less expanded products due to added wheat bran have been shown by Fleischman *et al*.^9^ The authors explained that the higher content of protein and fiber in wheat bran could have limited expansion of the snack.^9^ In fact, the expansion is related to the starch content, but also to other components that could interfere with it. Although in our samples the protein content was similar between the two raw materials (i.e., DWM and PSC), the fiber content was higher in DWM. Fiber can interfere with starch, hampering the expansion at the die exit, thus preventing the formation of air bubbles to their maximum size.^37^ The mechanistic effect for lower expansion is mostly due to the insoluble fiber, which changes the expansion by affecting the basic steps of the process: starch transformation, bubble nucleation, bubble growth, and bubble collapse.^38^ The first effect is due to the chemical composition of the insoluble fiber itself, which is less hydrophilic and less soluble than starch, so when it is added the extrudates end up with lower water solubility and water absorption.^38^ When keeping the total water content constant, adding more wheat bran has been shown to lower the glass transition temperature of the starchy matrix.^38^ This happens because fiber absorbs less water than starch does, so more of the total water stays available to the starch phase instead.^38^ Indeed, Robin *et al*.^38^ reported that the formation of a higher number of small bubbles is due to insoluble fibers, corroborating our results. Moreover, the poor interfacial adhesion between bran particles and the starch matrix results in weak interfacial regions that act as rupture points during expansion. Consequently, cell expansion is limited. This interpretation is supported by the X‐ray micro‐CT analysis, which revealed a progressive reduction in object volume, total porosity, and pore size with increasing DWM content, together with the formation of a more homogeneous and compact cellular structure.

**Table 2:** Physical and physicochemical properties of snacks produced from pea starch concentrate (PSC) and durum wheat meal arabinoxylan fraction (DWM)

Sample	ER (%)	Specific volume (mL g^−1^)	WHC (g water g^−1^ snack)	WSI (%)	OHC (g oil g^−1^ snack)
PSC	6.88 ± 0.63a	24.35 ± 1.32a	3.90 ± 0.16ab	39.74 ± 1.10a	2.57 ± 0.03a
DWM_5%	5.42 ± 0.36b	20.40 ± 0.49b	4.30 ± 0.30a	44.49 ± 2.30a	2.57 ± 0.03a
DWM_10%	4.38 ± 0.63bc	15.11 ± 0.23c	3.76 ± 0.13b	39.08 ± 2.27a	2.73 ± 0.09a
DWM_15%	3.54 ± 0.36 cd	12.74 ± 1.01 d	3.16 ± 0.26c	43.61 ± 2.19a	2.72 ± 0.09a
DWM_20%	2.92 ± 0.36de	12.91 ± 0.56d	3.23 ± 0.04c	39.45 ± 1.42a	2.70 ± 0.07a
DWM_25%	1.67 ± 0.36e	10.00 ± 0.72e	3.15 ± 0.11c	43.83 ± 3.47a	2.67 ± 0.03a

ER, expansion ratio; OHC, oil holding capacity; WHC, water holding capacity; WSI, water solubility index. Data are expressed as a mean ± SD (*n =* 3). Different letters in the same column indicate significant differences according to Tukey's HSD test (*P* ≤ 0.05).

Further discussion on this point is given in the section ‘Instrumental texture and color’, below. Moreover, Friero *et al*.^39^ have observed a lower expansion in wheat and barley AX extrudates, suggesting a significant influence of this portion of fiber in the ER. Indeed, a negative correlation was found in our samples between the ER and the AX content (Pearson's *r* = −0.989, *P* = 0.000; Supporting Information, Fig. S1). The second reason for the lower ER in DWM snacks was the higher amylose content in PSC. Thachil *et al*.^35^ found an increase in ER by increasing the amylose content (until it reached 50%), due to improvement of the gelatinization process during extrusion. In fact, a positive correlation was found between the ER and the amylose content (*r* = 0.922, *P* = 0.009; Fig. S1). Expansion and porosity are the most critical quality parameters for expanded snacks, as they determine consumer acceptability.^40^ Moreover, the degree of expansion influences the texture of extruded products, such as fragility, crispness, and hardness.^9^, ^37^ The close relationship between expansion and texture was confirmed by microstructural analysis, which showed that the lower ER of DWM‐containing snacks was associated with a denser cellular network. As a consequence of lower ER, the specific volume has also decreased. Between all the physicochemical properties, specific volume has been reported to have the highest correlation with sensory attributes,^41^ suggesting that the samples containing DWM could also have lower consumer acceptance.

The WHC of snacks was negatively influenced by the increase in DWM ratio. WHC represents the ability of the product to retain water per gram of snack.^4^ The increase in fiber content, accompanied by the decrease in starch, led to a decrease in WHC.^42^ Robin *et al*.^38^ pointed out that the changes in WHC are a physicochemical track of how the fibers modify the extrusion process. Indeed, a marked decrease in WHC has been reported for sugar beet pulp and pea hulls after extrusion,^38^ an effect attributed to the loss of cohesion in the fiber cell walls caused by the harsh mechanical conditions of the process. The decrease in WHC is a positive aspect, considering that snacks with high WHC would become soggy by absorbing water, whereas snacks with low WHC would remain solid and crunchy, and would have a longer bowl life. The latter is a characteristic preferred by most consumers in different food products, such as breakfast porridge or breakfast cereals eaten with milk.^43^, ^44^

The WSI value was unaffected by DWM in the formulation. WSI measures the amount of soluble compounds formed during the extrusion process, mainly due to starch gelatinization and dextrinization. In the present study, WSI values above 39% were observed across all samples, suggesting a comparable degree of starch conversion regardless of DWM inclusion.

The OHC also did not show any statistical differences among the samples. OHC reflects the absorption of oil per gram of product^4^ and is closely related to the polysaccharide chemical structure and availability of lipophilic groups.^45^ It can be hypothesized that DWM, and AX in particular, contributed to the snack OHC similarly to PSC. OHC is an important property, since extruded snacks are often spray‐coated with oil‐based flavorings to enhance their acceptability and taste. Oil coating has been previously described as a common treatment for this type of product.^40^

### Predicted glycemic index and antinutritional compounds

The results of HI and pGI of the extruded snacks are shown in Fig. 5. A progressive and statistically significant reduction in both parameters was observed as the percentage of DWM increased. Specifically, while the addition of 5% DWM did not result in significant differences compared to the control (PSC), a statistically significant reduction in terms of HI and pGI was already observed with DWM_10%, becoming more pronounced as the proportion of DWM increased. Samples with the highest percentages of DWM (20% and 25%) showed the lowest pGI values. These results are consistent with previous studies reporting reductions in pGI following the incorporation of fiber‐rich ingredients into different food matrices.^20^, ^46^, ^47^ For example, enriching plant‐based ice cream with oat okara, rich in β‐glucans and dietary fiber, reduced the pGI from 76.43 in the control sample to 67.36 and 64.49 in formulations containing 12% and 15% of oat okara, respectively. Similarly, adding xylooligosaccharides to spreadable goat cheese reduced pGI by approximately 20% in the experimental cheese formulations compared to the control. Similarly, baked goods enriched with orange peel flour showed the lowest pGI (of about 60.37), compared to the control (67.15). These findings support the current results, where increasing DMW incorporation progressively reduced both HI and pGI, highlighting the beneficial role of fiber‐rich ingredients in modulating starch digestibility and pGI, This effect can be mainly attributed to the higher content of AX in DWM. In fact, extrusion is well known to promote a higher degree of starch gelatinization and dextrinization, which negatively affects the glycemic index value by making glucose available more quickly.^10^ However, the addition of arabinoxylans can interfere with starch gelatinization, and it also reduces the total amount of starch. Scientific evidence has demonstrated the ability of AX to modulate blood glucose and lipid metabolism and to exert prebiotic functions both *in vitro* and *in vivo*.^36^ Lu *et al*.^48^ reported lower postprandial glycemic and insulin responses after consuming white bread enriched with 6 g of arabinose. Lu *et al*.^49^ indicated that supplementation with 15 g per day of AX extracted from wheat flour by‐products in people with type II diabetes could significantly improve glycemic responses. The inclusion of DWM, rich in fiber, significantly modifies the microstructure of the extrudate, as shown in the section ‘X‐ray micro‐CT’, below. In particular, the presence of fiber components during extrusion cooking interferes with the gelatinization processes of starch, promoting the formation of slower‐digesting fractions, and reducing the availability of starch *in vitro*.^50^

**Figure 5:**
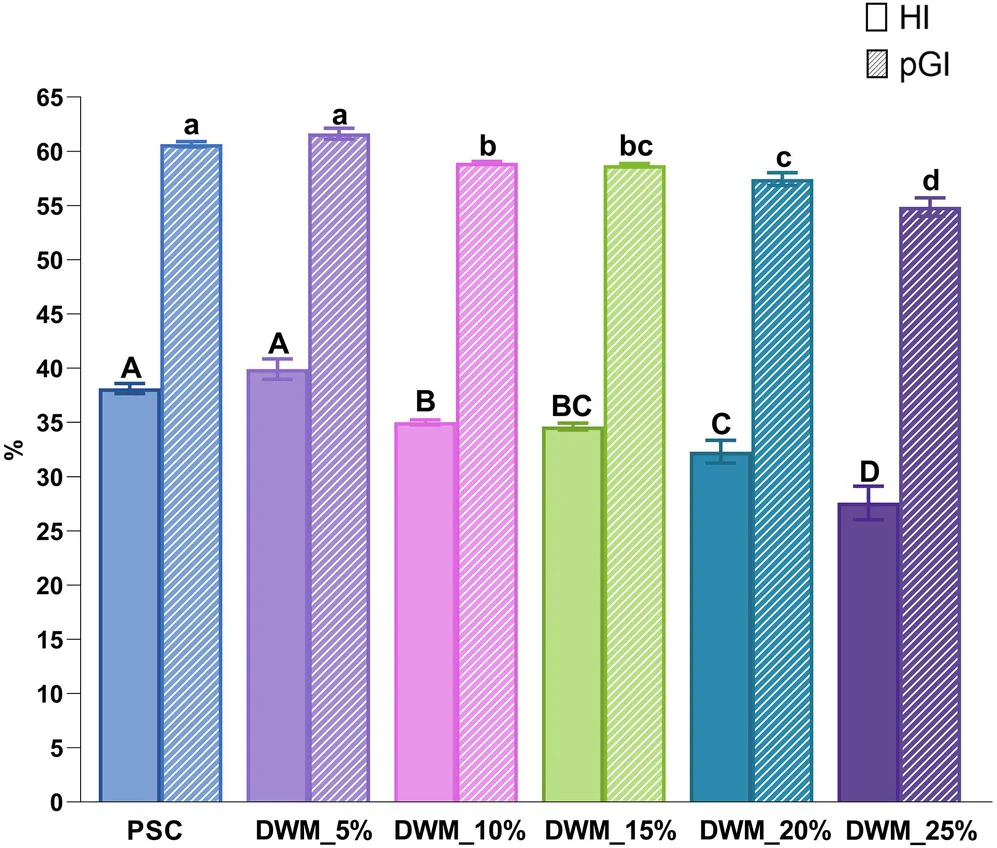
Results of hydrolysis index (HI) (solid color) and predicted glycemic index (pGI) (striped) of snacks produced from pea starch concentrate (PSC) and durum wheat meal arabinoxylan fraction (DWM). The values represent means of triplicates ± SD. Different uppercase letters indicate significant differences (*P* < 0.05) between the HI values according to one‐way ANOVA, followed by Turkey's HSD test; different lowercase letters indicate significant differences (*P* < 0.05) between the pGI values according to one‐way ANOVA, followed by Turkey's HSD test.

Overall, these findings suggest potential nutritional implications; however, since the glycemic index values reported here are based on an *in vitro* predictive model, *in vivo* studies are needed to confirm whether these predicted outcomes translate into actual physiological responses. Reducing the glycemic index of foods is an important goal not only for individuals with diabetes but also for the general population, as it can contribute to the prevention of chronic metabolic diseases.^51^

Table 3 presents the results of phytic acid and non‐digestible oligosaccharides and sucrose in expanded snacks. The phytic acid content was lowered by increasing the DWM ratio in the snack formulation. This reflects the composition of the raw materials, in which DWM contained 0.85 g 100 g^−1^ of phytic acid, whereas PSC contained 1.34 g 100 g^−1^. Compared with the raw materials, the thermomechanical treatment occurring during extrusion (e.g., high temperature, shear, and moisture), likely contributed to an observed reduction in phytate content.^8^ Phytic acid and oligosaccharides are antinutritional compounds found in legumes, cereals, and other plants. About 60–90% of phosphorus in cereals is in the form of phytic acid.^52^ Phytates inhibit some digestive enzymes and reduce the availability of positively charged micronutrients, such as Fe, Zn, Ca, and Mg, by chelation.^8^, ^52^

**Table 3:** Antinutritional compounds and sucrose content of snacks produced from pea starch concentrate (PSC) and durum wheat meal arabinoxylan fraction (DWM)

Sample	Phytic acid (g 100 g^−1^)	Verbascose + stachyose (mg g^−1^)	Raffinose (mg g^−1^)	Sucrose (mg g^−1^)
PSC	1.02 ± 0.04a	38.16 ± 0.72a	0.94 ± 0.11a	32.00 ± 2.50ab
DWM_5%	0.88 ± 0.09ab	35.99 ± 0.69a	0.57 ± 0.19b	32.41 ± 0.71a
DWM_10%	0.81 ± 0.02bc	28.61 ± 0.38b	n.d.	28.25 ± 1.33bc
DWM_15%	0.77 ± 0.02 cd	29.13 ± 1.02b	n.d.	26.83 ± 1.61c
DWM_20%	0.60 ± 0.13e	28.78 ± 0.79b	n.d.	29.31 ± 0.67abc
DWM_25%	0.66 ± 0.09de	29.37 ± 1.25b	n.d.	28.90 ± 0.34abc

PSC, 100% pea starch concentrate; n.d., not detected. Data are expressed as mean ± SD *(n* = 3). Different letters in a column indicate significant differences according to Tukey's HSD test (*P* ≤ 0.05).

The concentrations of verbascose + stachyose and sucrose were lowered by adding at least 10% of DWM, whereas raffinose decreased also in DWM_5%. Oligosaccharides, such as raffinose, verbascose, and stachyose, which are present in peas, are classified as non‐digestible because the enzyme α‐galactosidase is not produced by the human digestive tract. However, these oligosaccharides undergo fermentation by anaerobic microorganisms in the digestive tract, resulting in flatulence and digestive discomfort.^53^ For this reason, the consumer acceptability of legumes is limited, leading to lower consumption.^8^, ^53^, ^54^ However, several probiotic functions attributed to oligosaccharides, such as the suppression of pathogenic microorganisms, stimulation of bifidobacterial growth, enhancement of immune response, and potential protection against colon cancer,^53^ have led to a renewed interest in these compounds. Consequently, further investigation is needed to assess the digestibility of these products, carrying out *in vivo* studies.

### Instrumental texture and color

The texture and color of the snacks are presented in Table 4. Hardness increased with the increase in DWM content in the formulation, while crispness decreased. Hardness is defined as the maximum force needed to compress the extrudate, and it usually has an inverse relationship with ER.^55^ Crispness is related to the density of the porous structure of extrudates; indeed, a less dense structure leads to lower hardness and higher crispiness,^55^ as confirmed by a negative correlation between ER and hardness (Pearson's *r* = −0.970, *P* = 0.001; Supporting Information, Fig. S1) and a positive correlation between ER and crispness (*r* = 0.890, *P* = 0.018; Fig. S1).

**Table 4:** Instrumental texture and color of snacks produced from pea starch concentrate (PSC) and durum wheat meal arabinoxylan fraction (DWM)

Sample	Hardness (g)	Crispness	L*	a*	b*
PSC	2325 ± 164e	41.33 ± 4.23a	68.02 ± 0.50a	6.12 ± 0.04b	16.56 ± 0.44b
DWM_5%	2855 ± 286e	45.17 ± 10.34a	68.47 ± 0.28a	6.72 ± 0.18a	18.65 ± 0.24a
DWM_10%	4440 ± 472d	25.83 ± 2.93b	68.13 ± 0.41a	6.93 ± 0.12a	18.88 ± 0.51a
DWM_15%	5836 ± 271c	10.50 ± 1.05 cd	68.37 ± 0.42a	6.77 ± 0.26a	18.88 ± 0.46a
DWM_20%	7457 ± 367b	19.50 ± 3.89bc	68.45 ± 0.67a	7.07 ± 0.14a	19.59 ± 0.66a
DWM_25%	9577 ± 920a	8.40 ± 1.95d	67.75 ± 0.66a	7.07 ± 0.19a	19.82 ± 0.28a

Crispness – peak count; L*, lightness; a*, from green to red; b*, from blue to yellow; PSC, 100% pea starch concentrate. Data are expressed as mean ± SD (*n* = 3 for color, *n* = 8 for texture). Different letters in a column indicate significant differences according to Tukey's HSD test (*P* ≤ 0.05).

Thachil *et al*.^35^ reported a lower hardness and higher expansion in corn extrudate samples containing higher amylose content. In this study, a negative correlation was observed between amylose content and hardness (Pearson's *r* = −0.982, *P* = 0.000; Supporting Information, Fig. S1), which supports this hypothesis. AX were positively correlated with hardness (Pearson's *r* = 0.993, *P* = 0.000; Fig. S1) and negatively correlated with crispness (Pearson's *r* = −0.904, *P* = 0.014; Fig. S1), indicating that AX content may also have influenced the structural properties of the snacks. This interpretation is supported by the X‐ray micro‐CT analysis, which revealed that increasing DWM levels progressively reduced pore size, total porosity, and object volume while producing a more homogeneous and compact cellular structure. In fact, Fleischman *et al*.^9^ observed that a higher wheat fiber content corresponded to higher hardness values due to the formation of smaller pores.

Color analysis revealed that the color coordinates a* and b* showed a small but significant increase in the formulations containing DWM, regardless of the quantity used, compared with PSC, while L* remained unchanged. Similar values were found by other authors in extruded snacks produced from wheat bran.^9^ The color of wheat bran is given by the polysaccharides within the pericarp and aleurone cells, such as cellulose, lignin, arabinoxylans, glucomannans, and β‐glucan, which confer a brown aspect to the material, together with phenolic compounds oxidized to brown quinones.^56^ The significant differences in a* and b* values compared to the control were linked to yellow and red, both imputable to the yellow pigments (carotenoids) typically present in durum wheat.^57^

### Sensory properties

The sensory properties of the snacks are reported in Table 5. The overall odor intensity and sweet odor attributes were similar to the control PSC in all the formulations, except for DWM_25%. However, DWM_5% displayed similarity to the control PSC, both in hardness and in crispness value. Roasty odor in the samples with DWM was similar to PSC, except for the samples DWM_20% and DWM_25%. Raw material odor intensity was similar to PSC in all the samples. Odor is related to the combination of volatile compounds already present in the raw materials or newly formed during the thermo‐mechanical treatment.^58^ For example, volatile compounds with sweet and roasty perception can be developed as a consequence of the Maillard reaction.^59^ Moreover, DWM was dried after oil extraction; therefore, we could hypothesize that roastiness is a consequence of this process. No off‐odors were recorded.

**Table 5:** Sensory analysis of snacks produced from pea starch concentrate (PSC) and durum wheat meal arabinoxylan fraction (DWM)

Descriptor	PSC	DWM_5%	DWM_10%	DWM_15%	DWM_20%	DWM_25%
O.Overall intensity	5.10 ± 0.79b	5.35 ± 0.99ab	5.25 ± 0.72ab	5.55 ± 0.69ab	5.80 ± 0.77ab	6.10 ± 0.97a
O.Raw material intensity	3.45 ± 0.89a	3.60 ± 0.75a	3.90 ± 0.55a	3.90 ± 0.64a	3.90 ± 0.72a	3.70 ± 0.66a
O.Sweet	2.35 ± 0.93b	2.65 ± 0.93ab	2.50 ± 0.83ab	2.70 ± 0.80ab	3.00 ± 0.65ab	3.15 ± 0.67a
O.Roasty	3.00 ± 0.92b	3.30 ± 0.98ab	3.60 ± 0.82ab	3.65 ± 0.81ab	3.85 ± 0.67a	3.95 ± 0.89a
T.Overall intensity	5.90 ± 0.85b	6.00 ± 0.79b	6.85 ± 0.59a	6.80 ± 0.62a	6.75 ± 0.55a	7.20 ± 0.77a
T.Raw material intensity	3.95 ± 0.83a	3.90 ± 0.97a	4.20 ± 0.62a	3.75 ± 0.64a	3.75 ± 0.55a	3.75 ± 0.79a
T.Sweet	1.30 ± 0.86a	1.40 ± 0.75a	1.10 ± 0.72a	1.00 ± 0.56a	1.35 ± 0.81a	1.30 ± 0.86a
T.Roasty	3.65 ± 0.81d	3.85 ± 0.93 cd	4.55 ± 0.89bc	4.80 ± 0.89b	5.20 ± 0.89ab	5.70 ± 0.73a
T.Bitterness	1.00 ± 0.92c	1.00 ± 0.79c	1.40 ± 0.60bc	1.70 ± 0.98bc	2.15 ± 0.99ab	2.65 ± 0.75a
T.Astringent	1.95 ± 0.83b	1.90 ± 0.79b	2.65 ± 0.75a	2.65 ± 0.67a	3.00 ± 0.56a	3.20 ± 0.62a
X.Hardness	1.00 ± 0.00c	1.10 ± 0.31c	2.05 ± 0.51b	2.30 ± 0.57b	2.60 ± 0.75ab	3.10 ± 0.97a
X.Crispness	8.15 ± 0.67a	7.85 ± 0.67a	7.05 ± 0.69b	6.40 ± 0.94bc	6.05 ± 0.83 cd	5.50 ± 0.95d
X.Puffiness	8.00 ± 0.79a	7.55 ± 0.94a	5.65 ± 0.88b	4.70 ± 0.73c	4.05 ± 0.83 cd	3.45 ± 0.94d
X.Adhesiveness	5.00 ± 0.86d	5.35 ± 0.93 cd	5.65 ± 0.49bc	6.00 ± 0.46ab	6.30 ± 0.57a	6.40 ± 0.68a

O., sensory odor; T., sensory taste; X., sensory texture. Data are expressed as mean ± SD (*n* = 20).

Different letters in a column indicate significant differences according to Tukey's HSD test (*P* ≤ 0.05).

The taste perception of the samples revealed some variation in certain attributes. Specifically, the overall taste intensity, as well as roasty and astringent attributes, were enhanced by the increased DWM content in the snack formulation. Roasty taste increase was in line with the corresponding odor perception. Bitterness was higher only in the samples DWM_20% and DWM_25% compared to the control. Bitterness and astringency are frequently linked to wholegrain foods due to the presence of phenolic compounds, especially tannins, located in the bran layers.^60^, ^61^, ^62^ The samples, however, did not show high values of this descriptor, with the maximum for sample DWM_25% with a score of 2.65 on a scale from 0 to 9. None of the other attributes showed any differences from PSC, and no off‐tastes were recorded.

Sensory evaluation of texture revealed differences in each attribute. Hardness increased with increasing DWM ratio in the formulation, whereas crispness showed the opposite trend. The sensory results confirmed the observations highlighted by the instrumental evaluation of texture. A good correlation between sensory and instrumental hardness was also found by Liu *et al*.^63^ Puffiness value decreased with increasing DWM content above 10% in the formulation, while adhesiveness increased. Puffiness is related to the feeling of porous structure when biting through, and it was highly correlated with sensory crispness (Pearson's *r* = 0.994, *P* = 0.000; Supporting Information, Fig. S1), as was also observed in the instrumental texture and tomographic analyses. In fact, it has been reported that the size of pores in snacks and the break of air pockets play a pivotal role in crispness perception.^64^ Adhesiveness indicates the extent to which the sample adheres to the teeth when chewed.^64^ High adhesiveness is often a factor that causes low acceptance of expanded snacks;^65^ therefore, the addition of DWM could decrease consumer acceptability at higher concentrations. Future studies could investigate these aspects in a consumer test.

## CONCLUSIONS

In this study, the effect of AX fraction derived from by‐products of durum wheat milling on the properties of expanded snacks was assessed. Specifically, the increasing substitution of pea starch with DWM caused a decrease in WHC, while the ability to bind oil remained unaffected.

The physicochemical properties of the produced extrudates indicated their potential suitability for use in a wider list of applications. Besides eating as a snack, their low WHC makes them suitable for the preparation of different products such as porridge or breakfast cereals, without compromising crunchiness. The OHC of the snacks was also evaluated, and the results showed that the presence of DWM in the formulation did not affect this parameter, indicating that the ability to bind oil coatings was not compromised by DWM. WSI was also not affected by the addition of DWM.

Additionally, DWM had major impacts on structural properties, evidencing a decrease in the specific volume and expansion ratio. These results are related to the higher fiber content and lower amylose loading of DWM. Quantitative and qualitative information about these effects were provided by X‐ray micro‐CT analysis. Overall, the morphological and morphometric data provided clear evidence that DWM incorporation modifies the expansion behavior and internal architecture of the extrudates, with implications for both texture and product quality. The instrumental hardness and crispness were negatively affected by DWM due to the lower expansion and porosity, compared to the control PSC. Optimization of extrusion conditions, such as temperature, screw speed, or moisture content, could mitigate these effects and allow the development of fiber‐enriched snacks with improved structural and sensory properties. Additionally, the incorporation of additives such as modified starches and emulsifiers could be explored in future studies to enhance the structural integrity and textural properties of the snack. From a nutritional point of view, the use of DWM positively affected the glycemic index modulation due to increased arabinoxylans content.

The sensory results revealed an increase in roastiness, bitterness, and astringency, typical of wholegrain products; nevertheless, no off‐odors and no off‐tastes were detected.

The presence of AX in these snacks offers potential for future valorization through health‐related labeling. According to Commission Regulation (EU) No. 432/2012, the claim ‘Consumption of arabinoxylan as part of a meal contributes to a reduction of the blood glucose rise after that meal’ applies only to foods containing at least 8 g of AX‐rich fiber from wheat endosperm (≥60% AX by weight) per 100 g of available carbohydrates.

Although the composition of snacks was not characterized, it likely reflects that of the ingredient mix and could be estimated accordingly. Since the AX used here derives from wheat bran, the current claim cannot be directly applied; however, a specific European Food Safety Authority authorization could be sought. Given the known benefits of AX, a claim extension to bran‐derived AX appears feasible and represents a promising avenue for future product valorization.

## CONFLICT OF INTEREST

The authors declare no conflict of interest.

## AUTHOR CONTRIBUTIONS

Vittoria Latrofa: conceptualization; methodology; formal analysis; investigation; data curation; writing – original draft; writing – review and editing. Aleksei Kaleda: conceptualization; resources; methodology; formal analysis; investigation; data curation; writing – original draft; writing – review and editing. Carlo Porfido: formal analysis; visualization; data curation; writing – original draft; writing – review and editing. Giusy Rita Caponio: formal analysis; investigation; data curation; writing – original draft; writing – review and editing. Giuseppe Natrella: investigation; data curation; writing – review and editing. Davide De Angelis: conceptualization; methodology; data curation; supervision; writing – review and editing. Roberto Terzano: supervision; resources; project administration; writing – review and editing. Antonella Pasqualone: supervision; resources; project administration; writing – review and editing.

## ETHICS STATEMENT

This sensory analysis was conducted in accordance with the ethical principles outlined in the Code of Ethics of the World Medical Association (Declaration of Helsinki) and the Principles of Academic Ethics by Tallinn University of Technology. The research followed the protocols designed to protect the rights, privacy, and welfare of participants. Descriptive sensory analysis was conducted at TFTAK AS (Tallinn, Estonia) by an internal sensory panel. The assessments were made by ten participating assessors, who gave written consent to take part in the sensory analysis. Participation in the study was voluntary, and panelists could withdraw from the test at any time. The selected panelists were in good health, had no known allergies to the components, and were informed before the analysis about the purpose and procedures of the study. Participants were assured of the confidentiality of their data.

## Supporting information


**Figure S1:** Pearson's correlation analysis of the data. ER, expansion ratio; WHC, water holding capacity; WSI, water solubility index; OHC, oil holding capacity; TV, total volume; OV, object volume; OV/TV, percent object volume; St.Th., structure thickness (mean); St.Sp., structure separation (mean); OSOV, object surface to object volume ratio; Po.V., total volume of pore space; TP, total porosity percent; O., sensory odor; T., sensory taste; X., sensory texture.

## Data Availability

The data that support the findings of this study are available from the corresponding author upon reasonable request.
